# Flavonoid Accumulation Plays an Important Role in the Rust Resistance of *Malus* Plant Leaves

**DOI:** 10.3389/fpls.2017.01286

**Published:** 2017-07-18

**Authors:** Yanfen Lu, Qi Chen, Yufen Bu, Rui Luo, Suxiao Hao, Jie Zhang, Ji Tian, Yuncong Yao

**Affiliations:** ^1^Plant Science and Technology College, Beijing University of Agriculture Beijing, China; ^2^Beijing Key Laboratory for Agricultural Applications and New Techniques Beijing, China; ^3^Beijing Nursery Engineering Research Center for Fruit Crops Beijing, China; ^4^College of Food Science and Engineering, Beijing University of Agriculture Beijing, China; ^5^College of Horticulture and Landscape Architecture, Southwest University Chongqing, China

**Keywords:** abscisic acid, ethylene, jasmonate, salicylic acid, flavonoid biosynthesis, MYB transcription factor superfamily, rust, *Malus*

## Abstract

Cedar-apple rust (*Gymnosporangium yamadai* Miyabe) is a fungal disease that causes substantial injury to apple trees and results in fruit with reduced size and quality and a lower commercial value. The molecular mechanisms underlying the primary and secondary metabolic effects of rust spots on the leaves of *Malus* apple cultivars are poorly understood. Using HPLC, we found that the contents of flavonoid compounds, especially anthocyanin and catechin, were significantly increased in rust-infected symptomatic tissue (RIT). The expression levels of structural genes and MYB transcription factors related to flavonoid biosynthesis were one- to seven-fold higher in the RIT. Among these genes, *CHS, DFR, ANS, FLS* and *MYB10* showed more than a 10-fold increase, suggesting that these genes were expressed at significantly higher levels in the RIT. Hormone concentration assays showed that the levels of abscisic acid (ABA), ethylene (ETH), jasmonate (JA) and salicylic acid (SA) were higher in the RIT and were consistent with the expression levels of *McNCED, McACS, McLOX* and *McNPR1*, respectively. Our study explored the complicated crosstalk of the signal transduction pathways of ABA, ETH, JA and SA; the primary metabolism of glucose, sucrose, fructose and sorbitol; and the secondary metabolism of flavonoids involved in the rust resistance of *Malus* crabapple leaves.

## Introduction

*Gymnosporangium yamadai* Miyabe, which is known as Cedar-apple rust, causes serious diseases and significant economic losses to apple cultivars (Lee et al., 2016). As the disease progresses, infected leaves exhibit expanding leaf spot lesions, which lead to complete leaf collapse (Sekiyama et al., 2017). The resistance responses of plants under stress include a series of complex morphological, physiological and molecular process changes, such as osmotic adjustments, hormone regulation and resistant component formation (Vorwerk et al., 2004; de Torres Zabala et al., 2009; Lu et al., 2014; Tauzin and Giardina, 2014). Flavonoids are the most important plant pigments, and they are involved in UV filtration and symbiotic nitrogen fixation and can also act as chemical messengers, physiological regulators, and inhibitors against organisms that cause plant diseases, e.g., *Fusarium oxysporum* (Zhu et al., 2013; Zhou et al., 2014, 2017). Anthocyanins are an important subgroup of flavonoids and have been shown to act as a ‘sunscreen’ and protect cells from high-light damage by absorbing blue-green and ultraviolet light in photosynthetic tissues, thereby protecting the tissues from photoinhibition or high stress. This protective effect has been shown to occur in red juvenile leaves, autumn leaves, and broad-leaf evergreen leaves that turn red during winter (Jaakola, 2013; Zhu et al., 2013; Oglesby et al., 2016). Flavonols and proanthocyanidins have been suggested to play a role in suppressing the growth of *E. coli* bacteria and greatly reducing the ability of these bacteria to initiate an infection (Ge et al., 2013; Nguyen et al., 2016; Shang et al., 2017). In tissues, these substances may act as antioxidants and significantly contribute to the scavenging of free radicals produced via metabolic processes in plants, whereas at the organ surface, visible coloring phenotypes may be displayed because of the accumulation of flavonoids during exposure to pathogen infection or environmental stress. Visible coloring caused by flavonoids could represent a potential indicator of stress in plants for use in early diagnosis and prevention strategies; however, the coloring pattern of different species of plants is diverse and the underlying mechanisms are complex (Mierziak et al., 2014; Peixoto et al., 2016).

Flavonoid biosynthesis is an important branch of phenylalanine metabolism, and these compounds are regulated by the transcription profiles of *CHS, F3H, F3′H, DFR, ANS, UFGT* and *LAR* genes and manipulated by MYB transcription factors (Shi and Xie, 2014; Tian et al., 2015; Xu et al., 2015). This network in plants regulates the accumulation of flavonoid components, such as flavone, flavonol, flavanols, procyanidins and anthocyanins. When confronted with abiotic stress, the flavonoid composition in plant tissues and organs changes in response to possible cell damage, which induces changes in the coloring of visible parts (Cantín et al., 2007; Zhang et al., 2014). For example, plants often produce pigments around wounds, pathogen infection sites, insect piercing and feeding sites, and physical injuries, with various color responses observed among different plant varieties and even in different tissues (Petre et al., 2012; Dubovskiy et al., 2013; Fogelman et al., 2015; Yang et al., 2016). However, the role of these different color phenotypes caused by infection as well as the function of flavonoid composition changes and the mechanisms underlying the regulation of related gene transcription and their relationship to the metabolic pathways are not clear.

Osmotic regulation is a common physiological reaction in plants under stress. Carbohydrates are important components in the adjustment process, and many studies have shown that when plants experience drought, low temperature, high temperature and certain biological stresses, their tissues will accumulate substantial levels of fructose and sorbitol to maintain cell turgor (Couee et al., 2006; Lee et al., 2016). In addition, the accumulation of carbohydrates in stressed plants may be associated with the flavonoid biosynthesis pathway, and previous reports have demonstrated that high C/N promotes the accumulation of flavanols and anthocyanins in plants via the upregulated expression of structure genes and MYB10, MYB4 and MYB7 (Wan et al., 2015; Lu et al., 2017). We suggest that carbohydrates, which are a component of osmotic regulation during pathogen infection, may contribute to the accumulation of flavonoids as a defense against infection and disease expansion because of their antioxidant properties. The plant hormones ABA, ETH, JA and SA have multiple effects on secondary metabolism, including the flavonoid biosynthesis pathway (Das et al., 2012; Di et al., 2017; Zhu et al., 2017). For example, the study on grape berries demonstrated that ETH induced via the upregulated expression of the ACS gene regulated the accumulation of flavanols and anthocyanins by upregulating MYB transcription activities (Zhai et al., 2017). Recent study indicated that the accumulation of JA was stimulated by nematode infection together with LOX upregulation, which resulted in the accumulation of other second metabolites including flavonoids (Concha et al., 2013). ABA application in stressed plants have been shown to regulate many secondary metabolism pathways; thus, ABA may play an important role in regulating the expression of upstream genes in the flavonoid biosynthesis network (Cantín et al., 2007). SA is induced during biotic interactions and interact with JA and ETH-induced pathways, and play key roles in plant defense following pathogen attack (Robert-Seilaniantz et al., 2011; Di et al., 2017). However, the relationship of plant hormones and carbohydrates to the flavonoid biosynthesis network requires further exploration.

Under field conditions, we found that in the pathogen infection process of the ever-red-leaf cultivar ‘Royalty,’ obvious dark red spots appeared in infected tissues, and they corresponded with increased anthocyanin content. However, in the ever-green-leaf cultivar ‘Flame,’ yellow spots with unclear edges appeared because of obvious flavone and flavonol accumulation in the infected tissues. Therefore, the accumulation of flavonoids in two cultivars displayed obviously different coloration patterns when their leaves were infected by the rust pathogen, and even the uninfected and infected tissues of each cultivar presented differences in flavonoid metabolism. Therefore, we hypothesized that the flavonoid accumulation-based defense systems were different between the two cultivars.

To explore the differences in resistance between the two varieties and different leaf tissues, we measured the accumulation of flavonoids and the expression of key genes and transcription factors in the infected and uninfected tissues. We also investigated the expression of several key genes related to hormone metabolism and carbohydrate changes. Our aim was to explain the role of flavonoid biosynthesis in flavonoid-based defense mechanisms in the leaves of different varieties infected by rust fungus.

## Materials and Methods

### Plant Materials and Treatment

Eight-year adult trees of the purple-red cultivar ‘Royalty’ and green cultivar ‘Flame’ were used as experimental materials. Plants were obtained from the ornamental crabapple germplasm nursery of the Beijing University of Agriculture, which is located in the Changping District of Beijing, and they were managed under the same environmental conditions and infected by rust fungi under natural conditions (without artificial infection).

In the natural environment after the occurrence of rust, rust-infected leaves and normal leaves at the S1 (the early stage of rust infection with the scab obviously appeared in the leaf), S2 (the stage of aecidium appeared in the abaxial of the rust infected leaf), and S3 (the stage of aeciospore scattered in the rust infected leaf) rust developmental stages from 15 trees for each cultivar were sampled as one replicate for further tests. At least three replicates were performed for leaf collection. These samples were divided into 50 mL centrifuge tubes, frozen in liquid nitrogen, and then stored at -80°C.

### Determination of Flavonoid Content

Freeze-dried crabapple fruit samples were coarsely ground, and the powdered peel or flesh (0.8 g) from each sample was mixed with 10 mL of extract solution [methanol:water:formic acid:trifluoroacetic acid (TFA) = 70: 27: 2: 1] at 4°C in the dark for 72 h and shaken every 3 h. The liquid was then separated from the solid matrix via filtration through sheets of qualitative filter paper. The filtrate was further passed through 0.22 μm reinforced nylon membrane filters, and the filtrate was evaporated at 30°C and the residue was dissolved in 5 mL of water and purified by solid-phase extraction cartridge (500 mg, 3 ml) using C18 Supelclean ENVI-18 cartridge (Pretech Instruments, Sollentuna, Sweden). The cartridge was rinsed with water and methanol successively. Mobile phase A [TFA: formic acid: water (0.1: 2: 97.9)] and mobile phase B [TFA: formic acid: acetonitrile: water (0.1: 2: 48: 49.9)] were used to separate the anthocyanins from the flavonols via using an HPLC1100-DAD system (Agilent Technologies, Waldbronn, Germany). The gradients were as follows: 0 min, 30% B; 10 min, 40% B; 50 min, 55% B; 70 min, 60% B; and 80 min, 30% B. Anthocyanin detection was performed at 520 nm, and flavonol detection was performed at 350 nm. All samples were analyzed using three replicates.

### Determination of Soluble Sugar Content

Fresh samples (1 g DW) were extensively extracted three times in 5 mL 80% (v/v) ethanol at 80°C for 1 h. After extraction, the extracted liquid was mixed and evaporated to dryness in an evaporating dish over an 80°C water bath. The residues were re-dissolved in 1 mL ultrapure water and passed through 0.45 μM filters. Then, a 20 μL sample was injected into a HPLC system (Waters 600E) equipped with a carbohydrate column and an Alltech 2000ES evaporative light detector. A mixture of 80% ethanol and 20% ultrapure water (v/v) was used as the mobile phase, and the flow rate was set at 0.4 mL min^-1^. Fructose, glucose, sucrose and sorbital were identified and quantified from the retention times and peak heights of sugar standards purchased from Sigma. Each measurement was repeated three times.

### RNA Extraction, cDNA Synthesis and qPCR Assay

Total RNA was extracted from the samples using the RNAiso reagent according to the manufacturer’s instructions (TIANGEN). Highly pure RNA samples (5 μg) with a ratio of 260/280 nm > 1.9 were DNase treated (TaKaRa) and reverse transcribed into complementary DNA (cDNA) using an oligo(dT)_18_ primer and M-MLV reverse transcriptase (TaKaRa) following the manufacturer’s protocol. The synthesized cDNAs were then diluted to 4 ng/μL for the qPCR analysis. The qPCR assay (20 μL reactions: 9 μL SYBR^®^ Premix Ex Taq^TMII^, 2 μL 10 μM each forward + reverse primer, 7 μL water, and 2 μL of 4 ng/μL cDNA) was conducted on a CFX96^TM^ Real Time System (Bio-Rad). Differences in gene expression were calculated using the 2^(-ΔΔCt)^ analysis method, and the transcription levels were determined via relative quantification using the *Malus* 18S ribosomal RNA gene as the reference gene. The primers for qPCR were designed as described in **Table 1**.

**Table 1:** Oligo primer sequence used for quantitative real-time PCR to determine the expression of *Malus* cv. Royalty and Flame genes.

Accession number	Primer name	Primer sequence (5′-3′)	Experimental use
DQ341382	*18S-F*	GTCACTACCTCCCCGTGTCA	qRT-PCR
	*18S-R*	GAGCCTGAGAAACGGCTACC	qRT-PCR
JQ248934	*McPAL-F*	ACCCTGGACAGATTGAGGCAGCT	qRT-PCR
	*McPAL-R*	GCCTAGCGATCCTGCTTTGGCT	qRT-PCR
FJ599763	*McCHS-F*	TGACCGTCGAAGTTCGC	qRT-PCR
	*McCHS-R*	TTTGTCACACATGCGCTGGA	qRT-PCR
FJ817485	*McCHI-F*	AGGAGTTGTCGGAGTCCGTT	qRT-PCR
	*McCHI-R*	ACTTTCTCAGAGTATTGCTGGCC	qRT-PCR
FJ817486	*McF3H-F*	ACGAAGACGAGCGTCCAAAG	qRT-PCR
	*McF3H-R*	CTCCTCCGATGGCAAAGCAA	qRT-PCR
KF481684	*McF3′H-F*	CGTTGCTGTCGCTCACGGATGA	qRT-PCR
	*McF3′H-R*	ATGACGTGTCAGTGCCAGCTGTG	qRT-PCR
FJ817487	*McDFR-F*	CCGAGTCCGAATCCGTTTGT	qRT-PCR
	*McDFR-R*	CCTTCTTCTGATTCGTGGGGT	qRT-PCR
KF495602	*McFLS-F*	ACGAGCAACCGGGAATCACAACTG	qRT-PCR
	*McFLS-R*	CCCAGTTGGAGCTGGCCTCAGTA	qRT-PCR
FJ817488	*McANS-F*	CACAGGGGCATGGTGAACAA	qRT-PCR
	*McANS-R*	TTCACTTGGGGAGCAAAGCC	qRT-PCR
KF495603	*McUFGT-F*	TGGGCGGACACCAATCA	qRT-PCR
	*McUFGT-R*	ATGTCTCCACCGCACCA	qRT-PCR
KT276929	*McLAR-F*	TTTTGCCGTCGGAGTTTG	qRT-PCR
	*McLAR-R*	AGGGTGGGTGTTGTCAGAGTAG	qRT-PCR
KT276930	*McANR-F*	GCTGCTCCAGAAGGGCTAC	qRT-PCR
	*McANR-R*	TGGCTTTCACGCACGACT	qRT-PCR
KY111321	*McMYB4-F*	GACCAGCAGCAGAAACTA	qRT-PCR
	*McMYB4-R*	ACAACCCTCCATTAATGCCGAC	qRT-PCR
FJ817489	*McMYB7-F*	AGGGTGCAAAAACAGGCGCG	qRT-PCR
	*McMYB7-R*	CGGCACCCAGAAACCCCGAAC	qRT-PCR
JX162681	*McMYB10-F*	ACGCCACCACAAACGTCGTCG	qRT-PCR
	*McMYB10-R*	GGCGCATGATCTTGGCGACAGT	qRT-PCR
KP101181	*McMYBI6-F*	CACAACTCACAGGCCACTCA	qRT-PCR
	*McMYB16-R*	TGAAGCCCCAAACTGCAAGA	qRT-PCR
KC706480	*McLOX-F*	AAACTTCTGCAGCCTCATTTCC	qRT-PCR
	*McLOX-R*	AGGATTCCCCTGCCAATTG	qRT-PCR
EU716329	*McNCED-F*	CCCAAAACAACCCTGCAAC	qRT-PCR
	*McNCED-R*	AGACTAACGCTCCTTCGACCA	qRT-PCR
AB243060	*McACS-F*	TTTGATAGAGATTTGAGGTGGAGGA	qRT-PCR
	*McACS-R*	GTGGGTTTGATGGATTTGTGATTAG	qRT-PCR
MF280972	*McNPR1-F*	TCAAAAACAGAGCAGGGGCA	qRT-PCR
	*McNPR1-R*	GCGCAGCAAACTCAGATGTC	qRT-PCR

### Hormone Measurements via ELISA Assays

To quantify the content of ABA, JA, ETH and SA, 0.2 g fresh tissues samples were prepared for phytohormone extractions, and a hormonal analysis and quantification were performed using the enzyme-linked immunosorbent assay (ELISA) technique. The samples were first extracted with ethanol (70%, final concentration) and then were dissolved in phosphate buffer (0.02 M, Sigma, United States) and the ELISA assays for ABA, JA, ETH and SA were performed on a 96-well microtitration plate. After adding the hormone standards (1 mg/mL, Sigma, United States), sample extracts and antibodies (1 mg/mL, Sigma, United States), the coated plates were incubated for 40 min at 37°C. After rinsing four times, 100 μL peroxidase-labeled goat anti-rabbit immunoglobulin was added to each well, and then the plate was incubated for 40 min at 37°C. A colorimetric substrate (phenylenediamine) was added to each well, and the reaction was halted by the addition of 3 M H_2_SO_4_. Absorbance at 490 nm was detected using an ELISA spectrophotometer and used to calculate the ABA and JA content. Absorbance at 450 nm was detected using an ELISA spectrophotometer and used to calculate the ETH content. Each sample was measured three times with three biological replicates.

### Statistical Analysis

Significant differences among the experimental data were set to *p* = 0.05. The data were analyzed using a one-way ANOVA followed by Duncan’s and Tukey’s multiple range tests in Microsoft Excel 2010 and Data Processing System (DPS) software 7.05. The relationships between the data were analyzed using Pearson’s test with DPS software 7.05. Graphs were made with OriginPro 8 statistical software (OriginLab Corporation, United States), HemI 1.0 and Microsoft Office PowerPoint 2010.

## Results

### Phenotypes of Leaf Tissues Infected by Rust Disease (*Gymnosporangium yamadai* Miyabe) in the Two Cultivars

**FIGURE 1:**
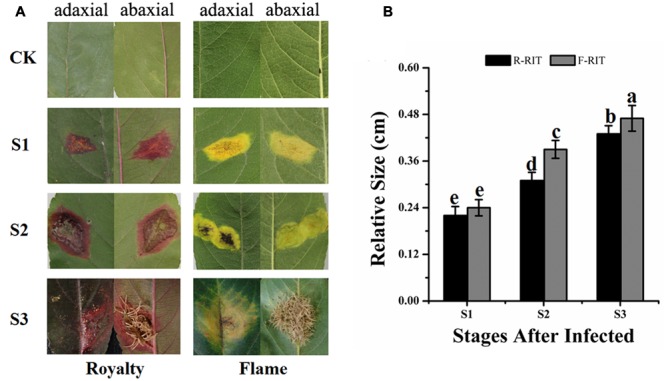
Expansion of rust spots in the leaf tissues of *Malus* crabapple cultivars infected by rust diseases. **(A)** Phenotypes of leaf tissues in *M.* cv. Royalty and Flame infected by rust diseases. **(B)** Expansion of rust spots in the leaf tissues. S1, S2 and S3 represent the different development stages after rust infection; and CK represents uninfected leaves. R-RIT and F-RIT represent rust spots in the leaf tissues infected by rust diseases in *M*. cv. ‘Royalty’ and *M*. cv. ‘Flame’, respectively. Error bars represent the means ± SD from at least three biological replications. a–e were calculated using one-way ANOVA followed by Duncan’s and Tukey’s multiple range tests.

We found that infected leaf tissues of different cultivars in an orchard displayed different color phenotypes during a rust disease outbreak in 2008–2010, with certain cultivars exhibiting red spots and the others showing yellow spots on the leaf surface. Rust spot areas on the infected leaves were obviously different among the pre-observed cultivars; therefore, we selected two typical cultivars, one with red spots and the other with yellow spots, to investigate the mechanisms underlying the different coloring of the rust spots and the differences between cultivars. We found that in the infected leaf tissues of the cultivar ‘Royalty,’ red patches with clear edges appeared at the front of the blade and hypha and red patches covered the back of the leaf, whereas in the infected leaf tissues of ‘Flame,’ yellow patches without a clear edge appeared on the front of the blade and hypha and yellow patches covered the back of the leaf (**Figure 1A**). The expansion size and expansion ratio of the spots during infection differed between the two tested cultivars and developmental stages. The patches in ‘Royalty’ were 0.22, 0.31, and 0.43 cm at S1, S2 (with an expansion ratio 40.91%), and S3 (with an expansion ratio 38.71%), respectively, whereas the patches in ‘Flame’ were 0.24, 0.39, and 0.47 cm at S1, S2 (with an expansion ratio 62.5%) and S3 (with an expansion ratio 20.51%), respectively (**Figure 1B**). Because of the coloration around the rust-infected area, we speculated that the two cultivars may have distinct resistance responses to rust infection based on flavonoid accumulation.

### Accumulation of Flavonoids in Leaf Tissues Infected by Rust Disease (*Gymnosporangium yamadai* Miyabe)

**FIGURE 2:**
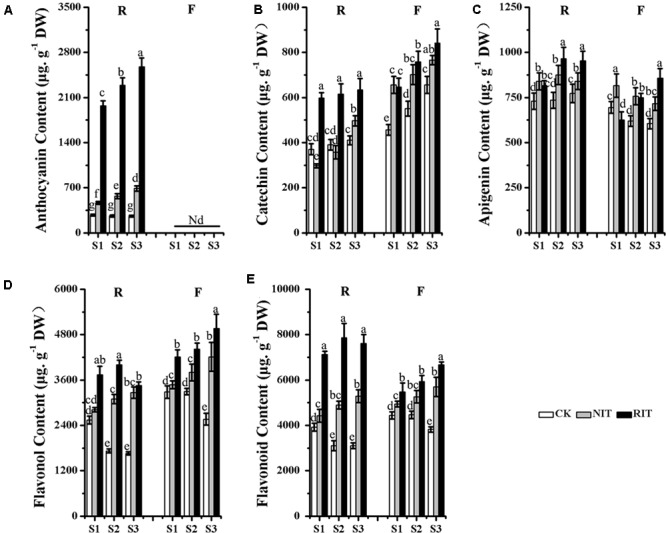
Contents of Anthocyanin **(A)**, Catechin **(B)**, Apigenin **(C)**, Flavonol **(D)** and Flavonoid **(E)** determined via HPLC analysis in the two cultivars *M*. Royalty and *M*. Flame at rust developmental stages. Significant differences among the experimental data were set to *p* = 0.05. a–g were calculated using one-way ANOVA followed by Duncan’s and Tukey’s multiple range tests in Microsoft Excel 2010 and Data Processing System (DPS) software 7.05. Error bars represent the means ± SD by at least three biological replications. R, Royalty; F, Flame; CK, normal leaf without rust infection; NIT, non-infected tissue of the leaf; RIT, rust-infected tissue.

‘Royalty’ had the highest levels of anthocyanin accumulation in the RIT during the disease spot development period, and the content of anthocyanin rose obviously and was significantly higher than that of the NIT and CK. In ‘Flame,’ no accumulation of anthocyanins was observed in the RIT, NIT or CK (**Figure 2A**). The catechin accumulation in the RIT of ‘Royalty’ was lower than that of ‘Flame’ at all stages. With the development of disease spots, the catechin content of the ‘Royalty’ RIT increased slowly, whereas the catechin content of the ‘Flame’ RIT increased quickly across the time points. The catechin content of the RIT was higher than that of the NIT and CK at all stages, whereas the catechin content of ‘Flame’ RIT was little lower than ‘Flame’ NIT at S1 (**Figure 2B**). The apigenin (the main components of flavone) content of ‘Royalty’ reached a higher level than that of ‘Flame’ from S1 to S3. With the expansion of disease spots, the content of apigenin in the RIT of ‘Royalty’ increased initially then decreased, whereas in the RIT of ‘Flame,’ it increased over the stages of disease spot expansion. The apigenin content of the NIT of ‘Royalty’ increased initially and then decreased, whereas in ‘Flame,’ it decreased across all stages. For the CK, the apigenin content in ‘Royalty’ increased across all stages at a lower accumulation ratio, and in ‘Flame,’ it decreased across all stages (**Figure 2C**). The flavonol accumulation in the RIT of ‘Royalty’ was lower than that of ‘Flame’ at all stages. With the development of disease spots, the flavonol content of the ‘Royalty’ RIT initially increased and then decreased, whereas the flavonol content of the ‘Flame’ RIT increased across the time points. The flavonol content of the RIT was higher than that of the NIT and CK at all stages (**Figure 2D**). The total flavonoid content in the RIT of ‘Royalty’ was higher than that of ‘Flame’ at S1, S2, and S3. With the development of disease spots, the total flavonoid content of the ‘Royalty’ RIT displayed an initially increasing and then decreasing trend, whereas that of ‘Flame’ increased overall, and the total flavonoid content in the RIT of the two cultivars were higher than that of the non-infected tissue (NIT) and control (CK) at the spot development stages (**Figure 2E**). The total flavonoid content was significantly negatively correlated to the expansion rate of the disease spot area (Supplementary Table S1).

The total flavonoid accumulation in the NIT of ‘Royalty’ was lower and appeared only slightly different in ‘Flame’ compared with that of the CK, and all types of flavonoids tested except for apigenin of ‘Royalty’ did not obviously accumulate in the peripheral tissues around the disease spots, thus indicating a self-resistance pattern to rust disease via pigment accumulation during rust spot expansion. These results indicated that the infected leaf tissues of ‘Royalty’ may adopt defense strategies that include the biosynthesis of anthocyanins, whereas ‘Flame’ might adopt defense strategies that include the accumulation of flavonol and flavanol to resist the expansion of rust disease.

### Expression Profile of the Structural Genes in Leaf Tissues Infected by Rust Disease (*Gymnosporangium yamadai* Miyabe)

**FIGURE 3:**
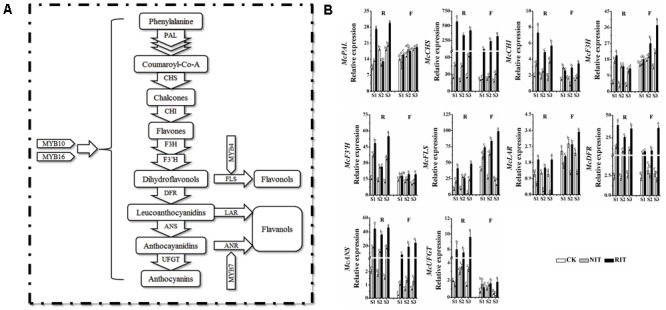
The flavonoid biosynthesis pathway **(A)** and relative expression profiles of flavonoid biosynthesis genes **(B)** in the two cultivars *M*. cv. Royalty and Flame. Significant differences among the experimental data were set to *p* = 0.05. a–i were calculated using one-way ANOVA followed by Duncan’s and Tukey’s multiple range tests in Microsoft Excel 2010 and DPS software 7.05. Error bars represent the means ± SD from at least three biological replications. R, Royalty; F, Flame; CK, normal leaf without rust infection; NIT, non-infected tissue of the leaf; RIT, rust-infected tissue of the leaf.

To explore the molecular mechanisms underlying flavonoid accumulation in rust-infected leaves, we analyzed the expression of key structural genes related to the flavonoid biosynthesis pathway (**Figure 3**). The results showed that multiple significant differences occurred in the expression of upstream genes, including *McPAL, McCHS* and *McCHI*, between the two varieties and their RIT and NIT. At each stage of the expansion of disease spots (S1, S2 and S3), the expression levels of *McPAL, McCHS* and *McCHI* in the RIT of ‘Royalty’ were higher than that of ‘Flame’ except for *McPAL* at S2. From S1 to S3, the expression levels of *McPAL, McCHS* and *McCHI* in the RIT of ‘Royalty’ initially sharply declined then increased slightly, and the expression of these genes in the RIT of ‘Flame’ increased obviously compared with their expression levels in the NIT and CK. The expression level of *McF3H* in the RIT of ‘Royalty’ was higher than that of ‘Flame’ only at S1. *McF3H* expression in the RIT of ‘Royalty’ displayed an initially sharp decline and then a slight increase, and in ‘Flame’ showed a sharply increasing trend. Compared with the expression of *McF3H, McF3′H* and *McFLS* in the NIT and CK, the expression of these genes in the RIT of the two cultivars was higher except for *McF3′H* and *McFLS* in ‘Royalty’ at S2. *McLAR* expression changed similarly in the CK and NIT of the two cultivars (initially increased then decreased for the CK and decreased over the stages of rust development for the NIT), whereas for the RIT, the expression in ‘Royalty’ initially decreased then increased and the expression in ‘Flame’ increased with rust development. For the downstream genes, including *McDFR, McANS* and *McUFGT*, their expression in ‘Royalty’ was higher than that in ‘Flame,’ and in the RIT, the genes displayed an initial large decrease then a small increase in ‘Royalty’ and showed significant increases in ‘Flame.’ In CK, the expression of these genes initially increased and then decreased in ‘Flame’ and initially decreased and then increased in ‘Royalty’ (except for *McANS*, which decreased with rust development). In the NIT, the expression of these genes initially increased and then decreased in ‘Flame’ (except for *McUFGT*, which decreased with rust development) and initially increased and then decreased in ‘Royalty’ (except for *McUFGT*, which initially increased and then decreased with rust development). The two cultivars may have differentially manipulated the transcription mechanism response to rust infection, with one initially activating transcription and subsequently increasing the biosynthesis pathways for anthocyanins and the other showing a linearly increasing stress response that promoted the levels of flavonol and flavanol biosynthesis.

### Transcription Level of MYBs in Leaf Tissues Infected by Rust Diseases

**FIGURE 4:**
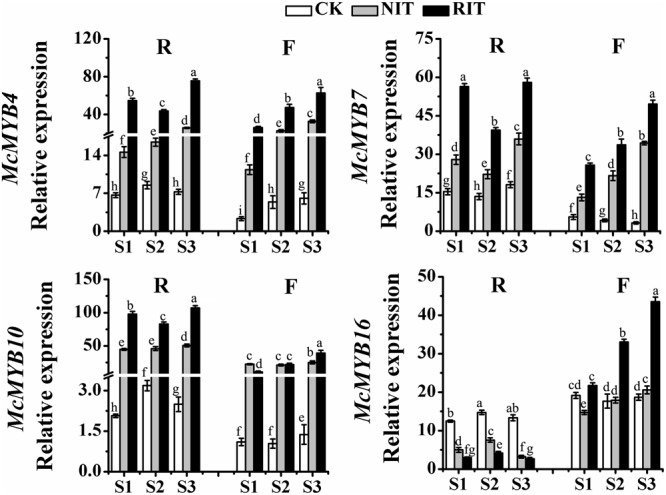
Relative expression profiles of MYBs in the two cultivars *M*. cv. Royalty and Flame at the rust developmental stages. Significant differences among the experimental data were set to *p* = 0.05. a–i were calculated using one-way ANOVA followed by Duncan’s and Tukey’s multiple range tests in Microsoft Excel 2010 and DPS software 7.05. Error bars represent the mean ± SD from at least three biological replications. R, Royalty; F, Flame; CK, normal leaf without rust infection; NIT, non-infected tissue of the leaf; RIT, rust infected tissue of the leaf.

Significant differences were observed in the expression of MYB transcription factors among the cultivars and the infected and uninfected leaf tissues (**Figure 4**). *McMYB7* and *McMYB10* expression in ‘Royalty’ was higher than that in ‘Flame,’ and the expression of these genes in the RIT was higher than that in the CK and NIT in both cultivars. *McMYB10, McMYB4* and *McMYB7* expression in the RIT of ‘Royalty’ had a V-type trend (initial decrease and then increase), whereas *McMYB16* expression had an opposite-shaped curve. Moreover, the expression of these genes in ‘Flame’ showed a linearly increasing trend with the expansion of rust spots, and the expression of these TFs in the RIT of ‘Royalty’ appeared to be lower at S2 than those in the NIT and/or CK. The expression of these four TFs except for *McMYB10* in the RIT of ‘Flame’ were higher than that in the NIT and CK. The MYB transcription factors participate in regulating flavonoid biosynthesis induced by rust infection and play an important role in the accumulation of anthocyanins as well as flavonol and flavanol in the infected sites and surrounding tissues of the two cultivars by manipulating the transcription of the main structural genes related to the flavonoid biosynthesis pathway.

### Accumulation of Carbohydrates in Leaf Tissues Infected by Rust Disease (*Gymnosporangium yamadai* Miyabe)

**FIGURE 5:**
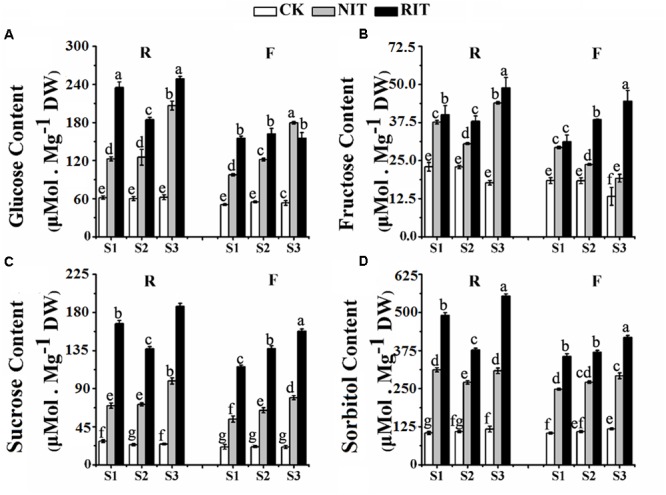
Measurements of the glucose **(A)**, fructose **(B)**, sucrose **(C)** and sorbitol **(D)** contents in the two cultivars *M*. cv. Royalty and Flame at the rust developmental stages. Significant differences among the experimental data were set to *p* = 0.05. a–g were calculated using one-way ANOVA followed by Duncan’s and Tukey’s multiple range tests in Microsoft Excel 2010 and DPS software 7.05. Error bars represent the means ± SD from at least three biological replications. R, Royalty; F, Flame; CK, normal leaf without rust infection; NIT, non-infected tissue of the leaf; RIT, rust-infected tissue of the leaf.

The accumulation of carbohydrates in plants under biotic and abiotic stress is defined as an osmotic regulation response, and it may also represent a resistance mechanism of plants to initial stress (Tauzin and Giardina, 2014; Lee et al., 2016). The total amount of carbohydrates in the RIT, NIT and CK of ‘Royalty’ leaves were higher than in ‘Flame’ (**Figure 5**). For ‘Royalty,’ the total amount and all carbohydrate components displayed a V-type trend with expansion of rust spots in the RIT, where the carbohydrate contents were higher relative to that of the CK and NIT. Moreover, glucose, sucrose and sorbitol accumulated to the same level in the CK, whereas fructose showed a slight decrease with rust development, and these contents in the RIT reached high levels compared with those in the NIT and CK, which indicates that osmotic regulation occurs in response to rust spot expansion. For ‘Flame,’ the total amount of carbohydrate, fructose, sucrose, and sorbitol in the RIT increased with the expansion of rust spots, whereas the amount of glucose did not change. The majority of carbohydrate components in the RIT had higher concentrations than that in the CK and NIT except for glucose.

### Expression of Genes Related Hormones in Leaf Tissues Infected by Rust Disease

**FIGURE 6:**
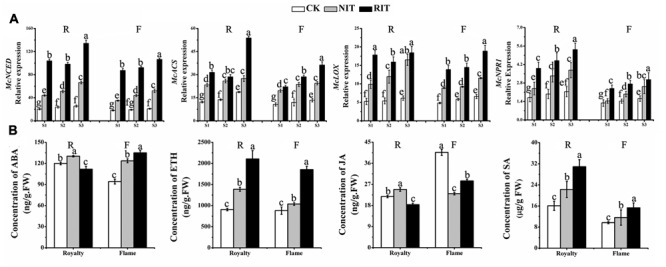
Relative expression profiles of hormone biosynthesis genes at the rust developmental stages determined via **(A)** qRT-PCR and hormones measured via **(B)** Enzyme-linked Immunosorbent Assay (ELISA) in the two cultivars *M*. cv. Royalty and Flame. Significant differences among the experimental data were set to *p* = 0.05. a–g were calculated using one-way ANOVA followed by Duncan’s and Tukey’s multiple range tests in Microsoft Excel 2010 and DPS software 7.05. Error bars represent the means ± SD from at least three biological replications. R, Royalty; F, Flame; CK, normal leaf without rust infection; NIT, non-infected tissue of the leaf; RIT, rust-infected tissue of the leaf.

Certain key genes in the ABA, ETH, JA and SA biosynthesis pathways were selected to investigate their expression and the subsequent hormone response to the expansion of rust spots. The results shown in **Figure 6A** indicate that significant differences occurred in the expression of three genes among the RIT, NIT and CK of the two cultivars: *McNCED*, which is a key gene of ABA biosynthesis (Zhang et al., 2015); *McLOX*, which is an important gene of JA biosynthesis (Christensen et al., 2014); *McACS*, which is a key gene of regulating ETH biosynthesis (Li et al., 2015); and *McNPR1*, which is an important SA-induced gene (Matern et al., 2015). *McACS* and *McNPR1* showed a significantly higher level of expression in the RIT than in the NIT and CK of the two cultivars at the rust expansion stages. The expression of these genes in the RIT of ‘Royalty’ showed a slight decrease and then increased with rust development, whereas their expression levels followed an increasing trend in ‘Flame.’ The expression of *McNCED, McACS*, and *McLOX* in the NIT and CK showed a significant increase with the expansion of spots in the two cultivars.

We measured the hormone concentrations of ABA, ETH, JA and SA at S3 in the two cultivars (**Figure 6B**). In ‘Royalty,’ the concentrations of ABA and JA in the RIT were lower than that in the NIT and CK, whereas ETH and SA showed the opposite trend. For ‘Flame,’ the concentrations of ABA, ETH and SA were higher in the RIT than in the NIT and CK, whereas JA was higher in the RIT than in the NIT but lower than that in the CK. ETH and SA were higher in ‘Royalty’ than in ‘Flame,’ whereas ABA and JA were lower in the RIT in ‘Royalty’ than in ‘Flame.’

These data indicated that key genes related with hormone biosynthesis and hormone concentration may be involved in the regulation mechanism of multiple pathways in the leaves infected by *Gymnosporangium yamadai* Miyabe.

### Analysis of Correlations among Structural Genes, TFs, Flavonoids, Hormones and Osmotic Substrates

**FIGURE 7:**
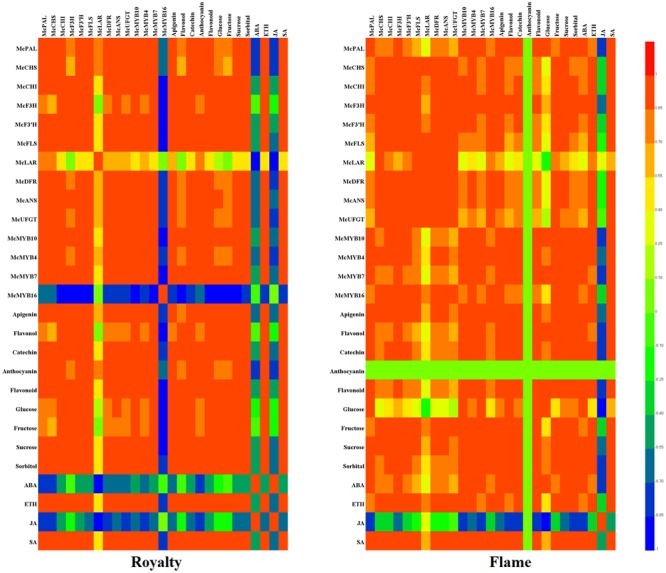
Heat map showing the correlation analysis between MYBs, flavonoids, osmotic substrates, hormones, and flavonoid biosynthesis structural genes in *M*. cv. Royalty and Flame. Correlation analysis were calculated using Pearson’s test with DPS software 7.05. The results calculated between –1 (blue color) and 1 (red color) are expressed in the colors shown on the right. All data represent at least three biological replicates.

In summary, primary metabolites (osmotic substrates) and secondary metabolites (flavonoids and hormones) are responsive to rust infection in leaves. We established a heat map showing the various correlations between flavonoid biosynthesis genes, MYBs, flavonoid compounds, hormone concentrations and osmotic substrates in the two cultivars (**Figure 7**).

The heat map indicates that the primary metabolites (glucose, fructose, sucrose, and sorbitol) had a positive effect on the secondary metabolites except for a negative effect on JA, and they also showed that most of the structural genes and MYBs had a positive effect on flavonoid biosynthesis except for *McMYB16*, which had a negative effect in ‘Royalty.’ In addition, most of the primary and secondary metabolites had a positive effect on rust resistance. Although ABA had a negative regulatory effect on others in ‘Royalty,’ ABA positively affected others for rust resistance in ‘Flame.’ Moreover, anthocyanins, *McMYB16* and ABA might be responsible for the different rust resistance phenomena in ‘Royalty’ and ‘Flame’ with rust infections.

## Discussion

### Coloring Response via Flavonoid Accumulation Can be an Indicator of Plant Tissues Primarily Impacted by Biotic Stress

When plants encounter biological and environmental stresses, they often manifest obvious local symptoms in their tissues and organs. For example, leaf parts infected by pathogens undergo the programmed death of cells and tissues, which leads to partial dehydration, blocks bacterial cells from organizing, and controls the spread of disease spots. Additionally, the piercing of plants parts by insects produces local changes, such as the corneous layer thickening, pastel encryption, etc., and these changes are known as direct defense responses of plants to insects and pathogens (Vorwerk et al., 2004; Kawamura et al., 2009; Mellway et al., 2009). In the complex response to biotic stress, spotted coloring in plants is an important symptom that indicates a visible direct defense to infection and possible defense and avoidance metabolism in plant tissues, and it also provides clues for control and management (Dubovskiy et al., 2013; Fogelman et al., 2015; Kawahigashi et al., 2016). Our results indicated that two cultivars of *Malus* crabapple exhibited pigment-related flavonoid accumulation in rust spots and the adjacent tissues, with ‘Royalty’ displaying red spots and ‘Flame’ displaying yellow spots because of an increase in anthocyanin and flavonol content, respectively. These increased contents were synchronized with spot expansion, and differences were observed between cultivars. These obvious coloring symptoms represent indicators of potential defense mechanisms, provide a signal for the degree and progress of pathogen infection, and generate comparative differences in stress resistance among cultivars.

### Different Coloring Patterns of the Two Cultivars Predicted Distinct Mechanisms of Pigment Accumulation under Biotic Stress

The accumulation of anthocyanins and flavonols is derived from the flavonoid biosynthesis pathway regulated by MYB transcription factors in plants, and this transcription network includes branches representing the metabolic pathways of dihydrogen chalcone, flavonols, flavanols and anthocyanins, which result in the variations in essential coloring diversity in the plant kingdom (Tian et al., 2015; Xu et al., 2015). Plants can initiate the whole network or parts of the network for defense, and different patterns of activation are observed when plants encounter biotic and environmental stress (Zhu et al., 2013; Zhou et al., 2014). Fogelman et al. (2015) reported that in the wound periderms of potato, the expression level of anthocyanin biosynthesis genes and regulators was downregulated, which resulted in a color change in the wounded periderm. The results of our flavonoid composition analysis indicated that different resistance patterns to rust disease occurred via pigment accumulation during the expansion of rust spots, In this stage, ‘Royalty’ may adopt strategies related to the biosynthesis of anthocyanins and ‘Flame’ may adopt strategies related to flavonol and flavanol accumulation to resist the expansion of rust disease. In the infected leaves of ‘Royalty,’ the transcription level of the genes in the flavonoid pathway exhibited an initial activation followed by a late increase, which increased the biosynthesis of anthocyanins, and in the infected leaves of ‘Flame,’ the transcription level displayed a linear upward trend for flavonol and flavanol biosynthesis. MYBs such as *McMYB10, McMYB4* and *McMYB7* showed a positive regulation effect, and whereas *McMYB16* displayed a negative effect, thereby activating different key genes in the same biosynthesis pathway of the infected leaves of the two cultivars. For example, the upregulation of *McCHS, McF3′H, McANS* and *McUFGT* in the infected spots of ‘Royalty’ and the upregulation of *McF3H, McFLS, McDFR* and *McLAR* in the infected spots of ‘Flame’ reflect the patterns of anthocyanin accumulation and flavonol accumulation, respectively (**Figures 2, 3, 7**).

### Carbohydrates and Hormones Are Involved in the Defense Response and Flavonoid Accumulation during Rust Spot Expansion

Carbohydrate accumulation plays an important role in the osmotic adjustment process of plants that are confronted with biotic and environmental stress. Many studies have shown that stressed plant cells accumulate pemoline, carbohydrates, polyamine, betaines, etc. (Couee et al., 2006; Lee et al., 2016). Our results indicated that the infected leaves in two both cultivars displayed a significant accumulation of carbohydrates, especially sucrose and sorbitol, which were associated with the accumulation of anthocyanins or flavonols and the expression of structure genes positively regulated by *McMYB4, McMYB7* and *McMYB10* and negatively regulated by *McMYB16* in the infected and adjacent tissues. Previous studies have demonstrated that high C/N promotes the accumulation of flavanols and anthocyanins in crabapple plants because of the high expression of structural genes and *McMYB10* (Wan et al., 2015; Su et al., 2016). The addition of sucrose had a positive effect on anthocyanin biosynthesis (Su et al., 2016). Therefore, carbohydrates, which represent an osmotic regulatory factor under pathogen infection, could contribute to the accumulation of flavonoids as a defense against spot expansion because of their antioxidant properties.

The plant hormones ABA, ETH and JA have been shown to demonstrate complex activity with regard to the flavonoid biosynthesis pathway (Zhou et al., 2009; Das et al., 2012; Zhai et al., 2017). Apple leaves infected with Alternaria activated flavonoid biosynthesis and the genes involved in phytohormone signaling pathways (ABA, JA, ETH) were upregulated (Zhu et al., 2017). Sugars and hormones can modulate anthocyanin biosynthesis via the coordinated regulation of the R2R3-MYB/bHLH/WD40(MBW) complex (Das et al., 2012).

All of these findings indicate that under rust infection, complicated regulatory cross-talk of the primary and second metabolites may occur in *Malus* cv. ‘Royalty’ and ‘Flame’.

### Regulation Network for Rust Resistance in Crabapple Leaves

**FIGURE 8:**
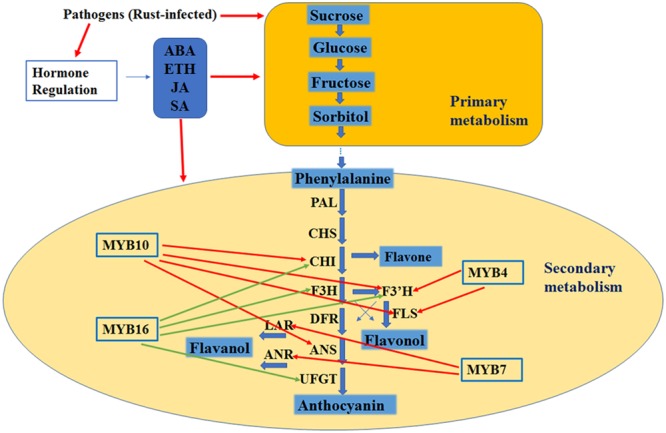
Hypothesis model of the regulation mechanism in leaves with rust infection. The red arrow indicates a positive effect, and the green arrow indicates a negative effect.

In our work to determine the resistance mechanisms of plants infected by rust disease, flavonoid biosynthesis structural genes and certain MYB transcript factors were activated, and this activation was accompanied by the accumulation of flavonoid contents. In addition, the accumulation of total carbohydrates and the four major carbohydrate components was higher in the rust-infected leaves, and the trends of the hormone biosynthesis genes and concentrations varied. Based on these data, we hypothesize that with rust infection, the primary metabolites were activated and then the secondary metabolites (flavonoid biosynthesis) were activated, and hormones were involved in this process by modulating the primary and secondary metabolites (**Figure 8**).

In summary, our results indicate that McMYB16 is a potential negative regulator and McMYB10 is a positive regulator of the leaf flavonoid accumulation in response to rust infection, and hormones (ABA, ETH, JA and SA) accumulation were induced in response to rust infection to regulate the primary and secondary metabolites directly and/or indirectly. Undoubtedly, the work described in this report is in accordance with the recent reports of Di et al. (2017) who suggested that the involvement of SA, ETH and JA signaling in tomato toward susceptibility against pathogen infect and Zhu et al. (2017) who suggested flavonoid biosynthesis were activated during apple leaves in response to *Alternaria alternata* apple pathotype infection this process. Our results provide insights on the flavonoid regulation network, which can be used to improve apple tree breeding and strategies for plant rust resistance.

## Author Contributions

YL, RL, and YY designed research. RL, QC, and YB performed research. YB, SH, JZ, JT, and YY analyzed data. YL, QC, YB, SH, and YY wrote the paper.

## Conflict of Interest Statement

The authors declare that the research was conducted in the absence of any commercial or financial relationships that could be construed as a potential conflict of interest. The reviewer PS-B and handling Editor declared their shared affiliation, and the handling Editor states that the process nevertheless met the standards of a fair and objective review.

## Abbreviations

ABA: abscisic acid
ANR: anthocyanidin reductase
ANS: anthocyanidin synthase
CHI: chalcone isomerase
CHS: chalcone synthase
DFR: dihydroflavonol 4-reductase
ETH: ethylene
F3′H: flavonoid 3′-monooxygenase
F3H: flavanone 3β-hydroxylase
FLS: flavonol synthase
JA: jasmonate
LAR: leucoanthocyanidin reductase
PAL: phenylalanine ammonia-lyase
RIT: rust-infected symptomatic tissue
SA: salicylic acid
UFGT: uridinediphosphate (UDP)-glucose: flavonoid 3-O-glycosyltransferase
