# Supplementary material for: Flavonoid Accumulation Plays an Important Role in the Rust Resistance of Malus Plant Leaves
Source: Front Plant Sci. 2017 Jul 18;8:1286. doi: 10.3389/fpls.2017.01286 (PMC5514348; doi:10.3389/fpls.2017.01286)

**Table S1** The negative correlation between the flavonoid content and the expansion rate of the disease spot area. R，Malus cv. Royalty; F, Malus cv. Flame; RIT, rust-infected tissue of the leaf. The data were performed from at least three biological replications.


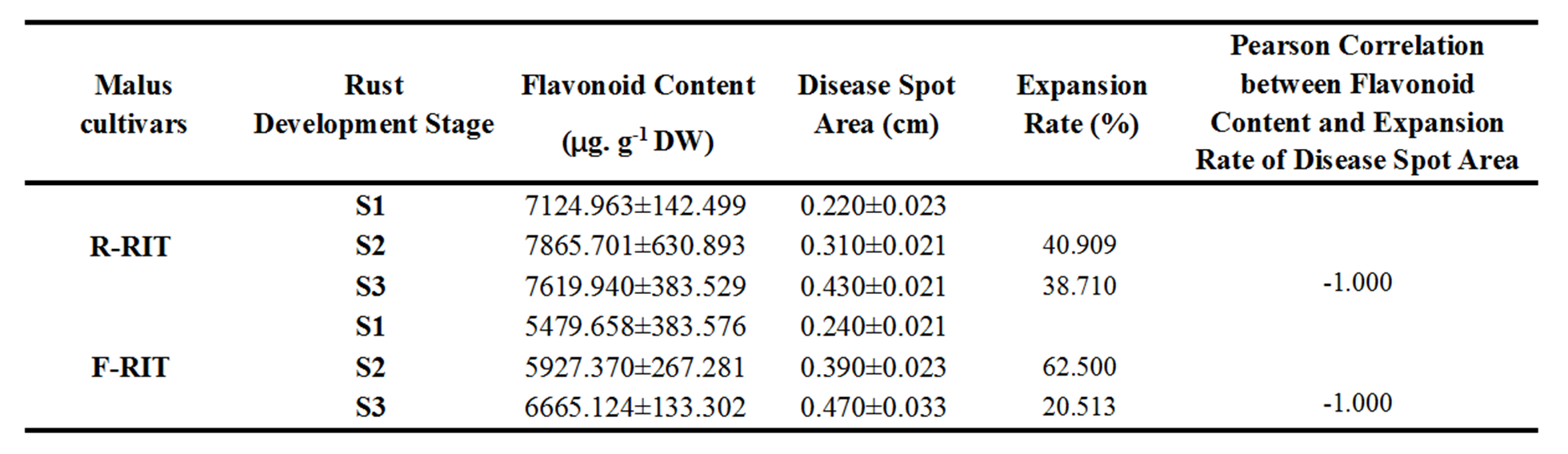

Supplement: Supplementary file 1 [file Table_1.DOC]
